# SMS121, a new inhibitor of CD36, impairs fatty acid uptake and viability of acute myeloid leukemia

**DOI:** 10.1038/s41598-024-58689-1

**Published:** 2024-04-20

**Authors:** Hannah Åbacka, Samuele Masoni, Giulio Poli, Peng Huang, Francesco Gusso, Carlotta Granchi, Filippo Minutolo, Tiziano Tuccinardi, Anna K. Hagström-Andersson, Karin Lindkvist-Petersson

**Affiliations:** 1 Department of Experimental Medical Science, Lund University, BMC C13, 221 84 Lund, Sweden; 2 Department of Pharmacy, University of Pisa, Pisa, Italy; 3 Department of Laboratory Medicine, Division of Clinical Genetics, Lund University, Lund, Sweden; 4 LINXS-Institute of Advanced Neutron and X-ray Science, Lund, Sweden

**Keywords:** AML, Acute myeloid leukemia, CD36, Fatty acid, Adipocyte, Cancer, Drug discovery, Molecular medicine

## Abstract

Acute myeloid leukemia (AML) is the most common form of acute leukemia in adults and the second most common among children. AML is characterized by aberrant proliferation of myeloid blasts in the bone marrow and impaired normal hematopoiesis. Despite the introduction of new drugs and allogeneic bone marrow transplantation, patients have poor overall survival rate with relapse as the major challenge, driving the demand for new therapeutic strategies. AML patients with high expression of the very long/long chain fatty acid transporter CD36 have poorer survival and very long chain fatty acid metabolism is critical for AML cell survival. Here we show that fatty acids are transferred from human primary adipocytes to AML cells upon co-culturing. A drug-like small molecule (SMS121) was identified by receptor-based virtual screening and experimentally demonstrated to target the lipid uptake protein CD36. SMS121 reduced the uptake of fatty acid into AML cells that could be reversed by addition of free fatty acids and caused decreased cell viability. The data presented here serves as a framework for the development of CD36 inhibitors to be used as future therapeutics against AML.

## Introduction

Bone marrow adipocytes expand with age and can take up more than 70% of the bone marrow volume^1,2^. They serve as reservoirs of energy that are stored in the form of triglycerides. Triglycerides can be broken down to glycerol and fatty acids (FA), in a process called lipolysis, and are exported out of the adipocytes to be used as energy by other cells. The bone marrow microenvironment is critical for the development of acute myeloid leukemia (AML) cells^3^, with the adipocytes providing energy for AML cell proliferation^4^. AML is characterized by abnormal proliferation of undifferentiated and nonfunctional hematopoietic blasts. These AML blasts have been shown to affect metabolic processes in adipocytes, by an unclear mechanism, and induce lipolysis enabling the transfer of fatty acids from adipocytes to AML blasts^5^. The imported fatty acids are broken down by fatty acid oxidation, which is crucial for AML cell survival^6^. Interestingly, it has been reported that leukemic stem cells also can use adipose tissue as a niche to support their increased metabolism, in particular those cells expressing the fatty acid translocase CD36, also called FAT or scavenger receptor class B member 3 (SCARB3)^7^. CD36 is known to facilitate the uptake of fatty acids over the cell membrane (long and very long chain fatty acids)^8^, and recently it was shown that very long chain fatty acid metabolism is essential for AML cell survival^9^. Thus, CD36 is a potential pharmacological target for inhibition of uptake of fatty acids in AML. This is further supported by evidence that AML patients with high CD36 expression have poor prognosis^10,11^. Combined, this suggests that CD36 is a valid drug target for AML. CD36 specific antibodies as well as fatty acid analogs have been reported to block the activity of CD36^12^, but what is clearly lacking are drug-like small molecule compounds targeting CD36 that can be developed in a cost effective manner into usable drugs for patients.

Here we have investigated the interplay between human primary adipocytes and AML cells and evaluated CD36 as a potential drug target. Upon co-culturing, fatty acids released from adipocytes were transferred to AML cells, possibly due to mechanisms involving the Inter-alpha-trypsin inhibitor heavy chain 1 (ITIH1) protein-induced lipolysis as indicated by mass spectrometry analysis. Moreover, a CD36 inhibitor was identified through receptor-based virtual screening studies and proven to block the activity of CD36 by combining surface plasmon resonance analysis with fluorescence microscopy. Finally, the viability of AML cells expressing CD36 was shown to be affected by exposure to the inhibitor.

## Methods

### Cell culture

AML cell lines THP-1 and KG-1 were obtained from DSMZ (Braunschweig, Germany). They were maintained at 37 °C in Gibco™ RPMI 1640 (Thermo Fisher Scientific, Waltham, MA, USA) with 1% penicillin/streptomycin and with or without (called starved cells) 10% fetal bovine serum (FBS). They were routinely checked for mycoplasma infection. Fresh media was added to cells the day before each experiment.

### Ethics

The study was approved by the Regional Ethics Committee, Regionala Etikprövningsnämnden in Lund (Dnr 2017/920). All experiments were performed in accordance with relevant guidelines and regulations and after written informed consent was obtained from all volunteers. Human abdominal subcutaneous adipose tissue was received from the Lund University hospital following surgery in female patients. The Declaration of Helsinki protocols were followed.

### Isolation of human primary adipocytes

Adipose tissue was obtained from three donors in total. The tissue was transported to the lab directly after surgery in phosphate buffer saline (PBS). Adipocytes (3 g per experiment) were isolated through excising fat lobules and cutting them into smaller pieces followed by digestion with collagenase at 37 °C, 170 rpm for 20 min and filtering through cotton mesh. Isolated cells were washed in Krebs–Ringer Hepes (KRH) buffer (118.6 mM NaCl, 4.7 mM KCl, 1.2 mM H_2_PO_4_, 1.2 mM MgSO_4_∙7H_2_O, 2.5 mM CaCl_2_∙2H_2_O, HEPES 25 mM pH 7.4, 2 mM glucose, 1% (w/v) bovine serum albumin (BSA), 200 nM adenosine). In the washing process the adipocytes, largely consisting of a lipid droplet, will float at the surface. The adipose stromal vascular fraction (SVF) sink to the bottom of the washing buffer, from where it can be removed by a syringe.

### Transfer of lipids from human primary adipocytes to AML cells

Isolated adipocytes were stained in KRH buffer containing 1 μg/mL C1-BODIPY 500/510-C12 fluorescent fatty acid analogue (#D3823, Thermo Fisher Scientific) for 1 h at 37 °C, 130 rpm. To remove excess stain, adipocytes were washed three times with KRH and three times with PBS (Cytiva Sweden, Uppsala, Sweden). Uptake of the fluorescent lipid by adipocytes and sufficient wash of excess lipid was verified with microscopy using the Axiovert 200 fluorescence microscope (Carl Zeiss Microscopy GmbH, Jena, Germany) with the acquisition software ZEN 2.5 (Carl Zeiss Microscopy GmbH) before they were transferred to RMPI 1640 media (10% FBS, 1% penicillin/streptomycin) using a double media change step. Adipocytes (1 mL floated, considered as 0.5 mL packed) were diluted with 4.5 mL RMPI 1640 media to a 10% (v/v) suspension from which 300 μL was added to a 24 well cell culture plate already containing 300 μL AML cells (150,000 cells/well), either THP-1 or KG-1. In control wells only media was added to AML cells. Lipids were allowed to transfer from adipocytes to AML cells for 22–24 h at 37 ℃ and 5% CO_2_. The quality of the adipocytes was inspected with light microscopy at the end of the co-culture to confirm that they were still intact. As the primary adipocytes mainly consist of a large lipid droplet, they float on top of the media in the well and can easily be removed from the samples by only collecting media and AML cells from underneath. Hence, the media with AML cells was collected with long, narrow pipette tips that were wiped off on the outside to remove contaminating adipocytes before transferring the AML cells to 1.5 mL tubes. To remove media and potential excess adipocytes, AML cells were spun down (5 min, 350×*g*) and the cell pellet was washed in PBS supplemented with 2% FBS two times before the AML cells were spun down onto microscopy slides and fixed in 4% paraformaldehyde (PFA) for 10 min. For washing, Tris buffered saline (TBS, 200 mM tris(hydroxymethyl)aminomethane, 150 mM NaCl, pH 7.6) with 0.1% Tween20 was used. Cells were permeabilized for 10 min (TBS, 0.25% Tween20) followed by subsequent washing steps. After blocking for 40 min in room temperature (RT) using blocking buffer (TBS with 0.1% Tween20 and 1% BSA) washing followed and incubation with primary antibody against beta-actin (1/1000, ab8227, Abcam, Cambridge, UK) in blocking buffer at 4 °C overnight. After washing the slides were incubated with secondary Alexa Fluor® 647 conjugated antibody (1/1000, A21244, Abcam) and 4',6-diamidino-2-phenylindole (DAPI, 1 µg/mL, Merck, Darmstadt, Germany) for 1 h in RT. Finally, slides were washed before being mounted for microscopy. Microscopy was performed on a Nikon A1 plus confocal microscope (Nikon, Amstelveen, The Netherlands) and pictures were obtained on NIS-elements, version: 4.50.02 (Nikon). Confocal stacks were assembled as maximum intensity projections and scalebars were added in ImageJ, version: 2.9.0/1.53t^13^.

### Mass spectrometry of media from AML cell and adipocyte co-culture

KG-1 cells were seeded into a 24 well plate (150,000 cells/well, 300 μL/well) in RPMI (10% FBS). Adipocytes were washed in KRH and RPMI 1640 before they were diluted to 10% (v/v) in RPMI 1640 (10% FBS). Adipocyte suspension (300 μL) was added to two wells with KG-1 cells and to two wells with RPMI 1640 media only. Two wells were kept with KG-1 and RPMI 1640 only. Cells were incubated for 20 h (37 °C, 5% CO_2_). The next day media was separated from the adipocytes and AML cells were removed by centrifugation (350×*g*, 5 min, 4 °C). The supernatants were flash frozen and stored in − 80 °C. Excess albumin from the FBS was removed with the Albumin Depletion kit (ab241023, Abcam) according to manufacturer’s protocol. Proteins were precipitated in 90% EtOH and 30 µg of protein sample was diluted in 100 mM ammonium bicarbonate to a final volume of 100 µL, reduced with dithiothreitol to a final concentration of 10 mM and heated at 56 °C for 30 min followed by alkylation with iodoacetamide to a final concentration of 20 mM for 30 min at room temperature in dark. The samples were precipitated overnight with ice cold ethanol to a final concentration of 90% ethanol. The samples were centrifuged at 14,000×*g*, for 10 min and the supernatants were removed. The samples were resolved in 50 µL of 100 mM ammonium bicarbonate and sonicated using a BioRuptor (40 cycles, 15 s on/15 s off). Digestion was performed by adding trypsin (Sequencing Grade Modified Trypsin, Part No. V511A, Promega, Madison, WI, USA) in a ratio of 1:50 (enzyme:protein) to the samples and incubated over night at 37 °C. The digestion was stopped by acidification with 10 µL 10% trifluoroacetic acid (TFA). The samples were dried by a centrifugal evaporator and the samples were resuspended in 0.1% TFA, 2% acetonitrile (ACN) to a peptide concentration of 0.25 µg/µL.

### Mass spectrometry acquisition

The LC–MS detection was performed on Tribrid mass spectrometer Fusion equipped with a Nanospray Flex ion source and coupled with an EASY-nLC 1000 ultrahigh pressure liquid chromatography (UHPLC) pump (Thermo Fischer Scientific). Peptides, 1 µg, was injected into the LC–MS. Peptides were concentrated on an Acclaim PepMap 100 C18 precolumn (75 μm × 2 cm, Thermo Scientific, Waltham, MA, USA) and then separated on an Acclaim PepMap RSLC column (75 μm × 25 cm, nanoViper, C18, 2 μm, 100 Å) at the temperature of 45 °C and with a flow rate of 300 nL/min. Solvent A (0.1% formic acid in water) and solvent B (0.1% formic acid in acetonitrile) were used to create a nonlinear gradient to elute the peptides. For the gradient, the percentage of solvent B was maintained at 3% for 3 min, increased from 3 to 30% for 90 min and then increased to 60% for 15 min and then increased to 90% for 5 min and then kept at 90% for another 7 min to wash the column. The Orbitrap Fusion was operated in the positive data-dependent acquisition (DDA) mode. The peptides were introduced into the LC–MS via stainless steel Nano-bore emitter (OD 150 µm, ID 30 µm) with the spray voltage of 2 kV and the capillary temperature was set 275 °C. Full MS survey scans from m/z 350–1350 with a resolution of 120,000 were performed in the Orbitrap detector. The automatic gain control (AGC) target was set to 4 × 10^5^ with an injection time of 50 ms. The most intense ions (up to 20) with charge states 2–5 from the full scan MS were selected for fragmentation in the Orbitrap. The MS2 precursors were isolated with a quadrupole mass filter set to a width of 1.2 m/z. Precursors were fragmented by high-energy collision dissociation (HCD) at a normalized collision energy (NCE) of 30%. The resolution was fixed at 30,000 and for the MS/MS scans, the values for the AGC target and injection time were 5 × 10^4^ and 54 ms, respectively. The duration of dynamic exclusion was set to 45 s and the mass tolerance window was 10 ppm. The raw DDA spectra were analyzed with Proteome Discoverer™ 2.5 Software (Thermo Scientific). Peptides were identified using SEQUEST HT against UniProtKB human database released 20,201,109 (canonical plus isoforms). The precursor and fragment tolerance were set to 15 ppm and 0.05 Da, respectively. Trypsin was selected as enzyme, methionine oxidation and acetylation of N-Terminus were treated as dynamic modifications and carbamidomethylation of cysteine as a fixed modification. Up to 2 missed cleavages were allowed and percolator was used for peptide validation at a q-value of maximum 0.01. Extracted peptides were used to identify and quantify them by label-free relative quantification. The extracted chromatographic intensities were used to compare peptide abundances across samples. Protein abundances were normalized against total amount of peptides. Statistics analysis was performed on GraphPad Prism 9.5.1 (GraphPad Software, San Diego, CA, USA). Normal distribution was assumed and a one-way ANOVA with Tukey’s multiple comparisons was used.

### CD36 expression in AML cells by microscopy

THP-1 and KG-1 cells were kept in PBS with 2% BSA and spun down onto microscopy slides. After fixation in 4% PFA for 10 min at RT and subsequent washing (TBS, 0.1% Tween20) cells membranes were permeabilized (TBS, 0.25% Tween20) and washed again. Cells were blocked, stained and visualized as described earlier with primary antibody against CD36 (1/1000, ab133625, Abcam), secondary antibody conjugated with Alexa Fluor® 488 (1/500, ab150061, Abcam) and DAPI (1 µg/mL, Merck). For control only secondary antibody and DAPI was used. Nikon A1 plus confocal microscope (Nikon) and NIS-elements, version: 4.50.02 (Nikon) was used for microscopy. ImageJ, version: 2.9.0/1.53t was used to add scalebars and change the Alexa Fluor® 488 signal to yellow, to not be confused with green lipids, by using LUTs yellow hot.

### Western blotting of CD36 expression in AML cells

THP-1 and KG-1 cells were spun down (350 xg, 5 min) and cell pellets were washed in cold PBS followed by another centrifugation step (2500×*g*, 10 min, 4 ℃). Cells were lysed during rotation in 4 ℃ for 10 min using 50 mM Tris–HCl (pH 7.4), 5 mM sodium pyrophosphate, 1 mM EDTA, 1 mM EGTA, 270 mM sucrose, 50 mM NaF, 1 mM DTT, 1% w/v NP-40, 50 μM sodium orthovanadate, and cOmplete™ protease inhibitor cocktail (Merck). Cell debris was removed by spinning at 14,000×*g* for 15 min at 4 ℃. Total protein concentration was determined with Bicinchoninic Acid Protein Assay Kit (Merck). Samples (14 μg) were run on a 4–12% Bis–Tris gel and blotted onto nitrocellulose membranes with wet transfer. Membranes were blocked with skim milk (10% w/v, 40 min, RT) and incubated at 4 ℃ overnight with primary antibodies diluted in TBS supplemented with 5% (w/v) BSA against CD36 (1/1000, ab133625, Abcam) and GAPDH (1/1000, sc-47724, Santa Cruz Biotechnology, Dallas, TX, USA). After washing (TBS, 0.1% Tween20) membranes were incubated in secondary corresponding HPR conjugated antibodies (1/5000, 31460 Invitrogen, Thermo Fisher Scientific and 1/5000, NA9310V, GE Healthcare, Chigaco, IL, USA) for 1 h at RT. Protein bands were visualized with SuperSignal™ West Pico Plus Chemiluminescent Substrate (Thermo Scientific).

### Uptake in AML cells of fluorescent fatty acid analogue in presence or absence of SMS121

To starve the AML cells, they were washed two times with RPMI 1640 without FBS by spinning down the cells (300×*g*, 5 min) and resuspension of the cell pellet before they were finally resuspended in RPMI 1640 without FBS and grown in 24 well plates (150,000 cells/well, 300 μL/well) for 21 h. SMS121 (Enamine Ltd, Kyiv, Ukraine) was diluted in DMSO to a stock concentration of 50 mM which was diluted in RPMI 1640 media in several steps and 300 μL was added to the AML cells at 0.2 mM concentration (0.4% DMSO). As control only DMSO was used. SMS121 was let to incubate for 50 min (37 ℃) before C1-BODIPY 500/510-C12 fluorescent fatty acid analogue (1 µg/mL, #D3823, Thermo Fisher Scientific) was added for additional 10 min. AML cells were collected and washed by two centrifugation (5 min, 350×*g*) and washing steps in PBS (2% FBS). Cells were spun down onto microscopy slides (4 min, 500 rpm) and fixed in 4% PFA in PBS (10 min, RT). After washing in TBS (0.1% Tween20) the cells were stained with DAPI in TBS (1/1000) for 10 min. Cells were washed in TBS (0.1% Tween20) before mounting. Microscopy was done on a Nikon A1 plus confocal microscope (Nikon) with NIS-elements, version: 4.50.02 (Nikon). Confocal stacks were combined as maximum intensity projections and scalebars added in ImageJ, version: 2.9.0/1.53t. Experiments were repeated two times in THP-1 cells and three times in KG-1 with 1–3 replicates/treatment.

### Docking calculations

With the aim of searching for new CD36 ligands using consensus docking, a hierarchical workflow was used to apply multiple docking procedures to a subset of the Enamine database comprising approximately 32,000 compounds endowed with at least one negatively charged moiety. All docking calculations were carried out using the X-ray structure of CD36 in complex with two palmitic acid molecules^14^. Twelve different docking procedures were used for this approach: Autodock 4.2.3, Dock 6.7, Glide 5.0 with the standard precision (SP) and extra precision (XP) method, GOLD 5.1 (with ChemScore, GoldScore, Astex Statistical Potential, and ChemPLP fitness functions), Autodock Vina 1.1, Glamdock 1.0, Plants, and rDOCK 1.0, employing previously described procedures^15,16^. For each docked ligand, the root-mean-square deviation (RMSD) of each docking pose relative to the remaining docking dispositions was calculated through the rms_analysis software from the Gold suite. By using an in-house program (unpublished), the different docking poses of each ligand were clustered, so that among the twelve results, all similar docking poses were grouped together. As a clustering algorithm, we used the complete-linkage method, which is an agglomerative type of hierarchical clustering. This method starts considering each element in a cluster of its own. The clusters are then sequentially combined into larger ones, until all elements are in the same cluster. At each step, the two clusters separated by the shortest distance are combined. We selected an RMSD clustering threshold of 2.0 Å, therefore, the so-obtained clusters contained groups of poses that are less than 2.0 Å away from all other poses belonging to the same cluster. All ligands showing a consensus level of at least eight (i.e., for which at least eight docking methods produced the same binding mode) were taken into account.

### Molecular dynamics simulations

All simulations were performed using AMBER 16. The complexes were placed in a rectangular parallelepiped water-box, an explicit solvent model for water (TIP3P) was used; the complexes were solvated with a 10 Å water cap. Before MD simulations, the whole system was energy-minimized through 5000 steps of steepest descent followed by conjugate gradient, until a convergence of 0.05 kcal/(mol·Å^2^), imposing a harmonic force constant of 10 kcal/(mol·Å^2^) only on the protein α carbons. The minimized complexes were used as starting conformations for the MD simulations. Particle mesh Ewald electrostatics and periodic boundary conditions were used in the simulation. The time step of the simulations was 2.0 fs with a cutoff of 10 Å for the non-bonded interaction, and SHAKE was employed to keep all bonds involving hydrogen atoms rigid. Constant-volume periodic boundary MD was carried out for 1 ns, during which the temperature was raised from 0 to 300 K. The system was then equilibrated through 34 ns of constant pressure simulation, using the Langevin thermostat to maintain the temperature of the system constant. Then, additional 65 ns of constant pressure MD production were performed, for a total of 100 ns MD simulation. All the α carbons of the protein were restrained with a harmonic force constant of 10 kcal/(mol·Å^2^) during the starting 35 ns of MD simulation. All the obtained MD trajectories were analyzed using the cpptraj program implemented in Amber 16.

### SMS121 synthesis

All the solvents and chemicals were used as purchased without further purification. Chromatographic separations were performed on silica gel columns by flash chromatography (silica gel pore size 60 Å, 40–63 μm particle size). Reactions were followed by thin layer chromatography (TLC) on Merck aluminum silica gel (60 F254) sheets that were visualized under a UV lamp. Evaporation was performed *in vacuo* (rotating evaporator). Sodium sulfate was always used as the drying agent. The proton (^1^H) and carbon (^13^C) NMR spectra were obtained with a Bruker Avance III 400 MHz spectrometer using the indicated deuterated solvents. Chemical shifts were given in parts per million (ppm) (δ relative to residual solvent peak for ^1^H and ^13^C). The ^1^H-NMR spectra were reported in this order: multiplicity and number of protons. Standard abbreviations indicating the multiplicity were used as follows: s = singlet, dd = doublet of doublets, t = triplet and m = multiplet.

### Abbreviations

EtOAc = Ethyl acetate; THF = Tetrahydrofuran; MeOH = Methanol; Hex = *n*-hexane; DMF = *N*,*N*-dimethylformamide.

### Synthesis of 3-methoxy-4-((2-methoxyethoxy)methoxy)benzaldehyde (2)

Sodium hydride (a 60% suspension of NaH in mineral oil, 1.3 eq) was added, under inert and dry atmosphere, to a solution of commercially available 4-hydroxy-3 methoxy benzaldehyde (**1**, 1 eq) in 16.4 mL of anhydrous THF at 0 °C. After 10 min, methoxyethoxymethyl chloride (1.6 equiv) was slowly added. The resulting mixture was kept under stirring for 2 h at room temperature. Then, the reaction mixture was placed in an ice bath (0 °C) and quenched with ice. The solution was repeatedly extracted with EtOAc. The organic phases were combined and then washed with a 1 N aqueous NaOH solution and a saturated water solution of NaCl, dried over Na_2_SO_4_, and the solvent was evaporated under reduced pressure to give the desired product (light yellow solid, 80% yield). Compound** 2** was used without further purification in the next step.

^1^H-NMR (CDCl_3_) δ(ppm): 3.63 (s, 3H); 3.53–3.57 (m, 2H); 3.85–3.89 (m, 2H); 3.94 (s, 3H); 5.42 (s, 2H); 7.31–7.34 (m, 1H); 7.41–7.45 (m, 2H); 9.88 (s, 1H).

### Synthesis of (E)-1-(4-(dimethylamino)phenyl)-3-(3-methoxy-4-((2-methoxyethoxy)methoxy) phenyl)prop-2-en-1-one (3)

Compound **2** (1 eq) was added to a solution of 1-(4-(dimethylamino)phenyl)ethan-1-one (1 eq) in 2.5 mL of MeOH. Then, solid NaOH (2.5 eq) was added, and the mixture was kept for 16 h at room temperature under stirring. The pH of the reaction mixture was brought to neutrality upon addition of a saturated solution of NH_4_Cl and then it was extracted with EtOAc. The organic layer was washed with a saturated water solution of NaCl and dried over Na_2_SO_4_. Then, the solvent was evaporated under vacuum. The resulting residue was purified by flash column chromatography (silica gel, eluent mixture Hex/EtOAc 7:3), to afford pure compound **3**, as bright yellow solid (73% yield).

^1^H-NMR (CDCl_3_) δ(ppm): 3.08 (s, 6H); 3.37 (s, 3H); 3.54–3.58 (m, 2H); 3.86–3.89 (m, 2H); 3.94 (s, 3H); 5.37 (s, 2H); 6.72 (d, 2H, *J* = 9.0 Hz); 7.16 (s, 1H); 7.20–7.24 (m, 2H); 7.46 (d, 1H, *J* = 15.4 Hz); 7.73 (d, 1H, *J* = 15.4 Hz); 8.00 (d, 2H, *J* = 9.0 Hz).

### Synthesis of (E)-1-(4-(dimethylamino)phenyl)-3-(4-hydroxy-3-methoxyphenyl)prop-2-en-1-one (4)

A 1 N aqueous solution of HCl (3.4 mL) was added to a solution of **3** (1 eq) in MeOH (3.4 mL), and the mixture was heated to 75 °C and kept under stirring for 1.5 h at the same temperature. Then, after cooling the reaction mixture to room temperature, a saturated solution of NaHCO_3_ was added to neutralize the solution. Usual workup (extraction with EtOAc, drying over Na_2_SO_4_ and the solvent evaporation) gave the desired product (light brown solid, > 99% yield). Compound **4** was used without further purification in the next step.

^1^H-NMR (CDCl_3_) δ(ppm): 3.08 (s, 6H); 3.96 (s, 3H); 5.89 (s, 1H); 6.71 (d, 2H, *J* = 8.8 Hz); 6.95 (d, 1H, *J* = 8.2 Hz); 7.13 (s, 1H); 7.22 (d, 1H, *J* = 8.2 Hz); 7.44 (d, 1H, *J* = 15.4 Hz); 7.73 (d, 1H, *J* = 15.4 Hz); 8.00 (d, 2H, *J* = 9.0 Hz).

### Synthesis of Ethyl (E)-2-(4-(3-(4-(dimethylamino)phenyl)-3-oxoprop-1-en-1-yl)-2-methoxyphenoxy)acetate (5)

Under inert and dry atmosphere, anhydrous K_2_CO_3_ (1 eq) was added to a solution of compound **4** (1 eq) in 1.7 mL of anhydrous DMF. After 20 min, ethyl bromoacetate (1.2 eq) was added, and the reaction mixture was kept for 16 h at room temperature under stirring. After dilution with water and usual workup (extraction with EtOAc, washing wityh brine, drying over Na_2_SO_4_ and the solvent evaporation) a crude product was obtained and purified by flash column chromatography (silica gel, eluent mixture Hex/EtOAc 6:4), to afford pure compound **5** as a yellow solid (92% yield).

^1^H-NMR (CDCl_3_) δ(ppm): 1.29 (t, 3H, *J* = 7.2 Hz); 3.09 (s, 6H); 3.96 (s, 3H); 4.27(q, 2H, *J* = 7.2 Hz); 4.73 (s, 2H); 6.72 (d, 2H, *J* = 8.9 Hz); 6.81 (d, 1H, *J* = 8.8 Hz); 7.16–7.21 (m, 2H); 7.46 (d, 1H, *J* = 15.4 Hz); 7.72 (d, 1H, *J* = 15.4 Hz); 8.00 (d, 2H, *J* = 9.0 Hz).

### Synthesis of (E)-2-(4-(3-(4-(dimethylamino)phenyl)-3-oxoprop-1-en-1-yl)-2-methoxyphenoxy) acetic acid (SMS121)

Compound **5** (1 eq) was solubilized in 1.5 mL of MeOH/THF (1:1 v/v). Then, the solution was treated with LiOH 2 N (6 eq), and the resulting mixture was kept for 2 h under stirring at room temperature. After neutralization with a saturated aqueous solution of NH_4_Cl a yellow solid precipitated. The suspension was filtrated and washed repeatedly with CHCl_3_ and cold water. The solid was collected and dried using a vacuum pump to give the desired final product (yellow solid, 82% yield).

^1^H-NMR (CD_3_OD) δ(ppm): 3.10 (s, 6H); 3.94 (s, 3H); 4.56 (s, 2H); 6.79 (d, 2 H, *J* = 9.0 Hz); 6.93 (d, 1 H, *J* = 8.2 Hz); 7.27 (dd, 1H, *J* = 8.3, 2.0 Hz); 7.38 (d, 1H, *J* = 2.0 Hz); 7.64–7.73 (m, 2H); 8.05 (d, 2H, *J* = 9.0 Hz).

^13^C-NMR (CD_3_OD) δ(ppm): 40.15; 56.56; 68.65 (2C); 112.02 (2C); 112.20; 114.29; 121.12; 124.03; 126.69; 130.24; 132.17(2C); 144.34; 150.86; 151.44; 155.44; 175.27; 190.18.

### Surface Plasmon Resonance (SPR) analysis

Interaction analyses were performed using a BIAcore 3000 (Biacore AB, Uppsala, Sweden) at 298 K. Biotin CAPture Kit (Cytiva Sweden, Uppsala, Sweden) and biotinylated human CD36, His, Avitag (CD6-H82E9) (Newark, DE, USA) were purchased. Before use, the CAP chip was conditioned and stabilized according to the manufacturer’s instructions. Immobilization buffer PBS (20 mM phosphate buffer, 150 mM NaCl, pH 7.4) and running buffer PBS supplemented with 5% DMSO were filtered and degassed, prior to use. Analyte stock solution at 2 mM concentration was diluted to a final concentration of 50 µM in running buffer and two-fold factor dilutions were made. During each binding cycle, the sensor chip was coated by the oligo-streptavidin solution (biotin CAPture reagent) by a 5-min injection at 2 µL/min. Biotinylated CD36 was captured on the flow cell at 20 µg/mL in the immobilization buffer at 5 µL/min at approximately 650 RU. The analyte (SMS121) was analyzed at a flow rate of 30 µL/min at five concentrations. Analysis was performed in duplicates, from the lowest to the highest concentration. The chip was regenerated in Guanidine-solution (8 M guanidine-HCl and 1 M NaOH, 3:1 v/v).

Data collected on the BIAcore 3000 were processed and analyzed using BIAevaluation software (version 4.1). Only reference subtracted data were considered. The average response of the last 10 s of the association for each cycle was calculated and plotted against the concentration to calculate the affinity constant, fitting the data to a steady state affinity with 1:1 interaction model (equation used was K_A_*Conc*R_max_/(K_A_*Conc*n + 1)).

### Concentration dependent inhibition of lipid uptake by SMS121

KG-1 cells were grown in a 24 well plate (300 µL, 150,000 cells/well) in RPMI 1640 without FBS for 24 h. A dilution series of SMS121 (Enamine Ltd) in DMSO was prepared with a starting concentration of 500 mM. Further dilution was done in RPMI 1640 and finally 300 μL of SMS121 dilution was added in triplicates to the wells giving concentrations of SMS121 in wells ranging from 0 to 500 µM (0, 62.5, 125, 250, 500 µM) with 0.1% DMSO. After 50 min incubation with SMS121 at 37 ℃, C1-BODIPY 500/510-C12 fluorescent fatty acid analogue (1 µg/mL, #D3823, Thermo Fisher Scientific) was added for an additional 10 min after which the samples were collected. After washing in PBS (2% FBS) and fixation as described previously, the cells were stained with DAPI (1/1000) and mounted with coverslips followed by microscopy on a Nikon A1 plus confocal microscope (Nikon) with NIS-elements, version: 4.50.02 (Nikon). Fluorescence intensities of taken up lipid per number of cells after each compound concentration was measured in ImageJ (version: 2.9.0/1.53t). From maximum intensity projections in grayscale the threshold was set using Otsu. Size exclusion for the DAPI channel was set to 10–600 and for the BODIPY channel to 0–600. Summary of each fluorescence in the BODIPY channel was divided by the number of nuclei determined by the DAPI channel.

### Cell viability by ATP assay

KG-1 and THP-1 cells were seeded into a white luminescence 96 well plate (25,000 cells/well, 50 µL). SMS121 (Enamine Ltd) was diluted in DMSO to a 1000 × stock concentration of 400 mM. From here a dilution series was made by diluting the compound in DMSO. Each concentration was then diluted further in media several times before being added in 50 µL volumes on top of the AML cells in triplicates, keeping the DMSO concentration in the wells at 0.1%. As control, DMSO was diluted similarly and added to the cells. The plate was incubated for 72 h (37 ℃, 5% CO_2_) before cell viability was determined by an ATP assay (CellTiter-Glo® 2.0, Promega) consistent with manufacturer’s protocol. Briefly, the plate was let to equilibrate for 30 min (RT) after which 100 µL of the reagent was added to the wells. After a short mixing step (300 rpm, 2 min) luminescence was measured using the Veritas™ Microplate Luminometer (Turner biosystems, Sunnyvale, CA, USA). From a total of four assays, the dilution series of one representative curve is shown. Effect on cell viability was calculated by decreasing luminescence levels after addition of SMS121, in comparison to the DMSO. IC_50_ was calculated with GraphPad Prism 9.5.1 (GraphPad Software, San Diego, CA, USA).

### Cell proliferation and viability by trypan blue exclusion assay

KG-1 and THP-1 cells were seeded in a 96 well plate (40,000 cells/well, 75 µL) in RPMI 1640 media with 2% FBS. For rescue experiments either 150 µM Oleic acid in PBS with 850 µg BSA (O3008, Sigma-Aldrich, Saint Louis, MO, USA), or PBS with 850 µg fatty acid free BSA (Thermo Fisher Scientific) was added to the wells. SMS121 (Enamine Ltd) was diluted in DMSO. It was further diluted 1000 × in RPMI 1640 media with 2% FBS before being added to cells at the final concentration of 150 µM. DMSO control was treated likewise and the final DMSO concentration in all wells was 0.1% and the total well volume 167 µL. Each treatment was done in triplicate wells and the experiment was done twice. Cells were incubated for 96 h (37 ℃, 5% CO_2_). Number of viable cells were counted on the Countess II FL automated cell counter (Thermo Fisher Scientific) according to manufacturer’s protocol. Shortly, the cells were mixed with a pipette and 10 µL cells were added to 10 µL trypan blue. Live number of cells/mL was recorded for each sample at 30 s after trypan blue addition. Graphs showing percentage of live cells for SMS121 treated cells compared to DMSO treated cells, were made in GraphPad Prism 9.5.1. A two-tailed unpaired t-test was performed comparing the differences of means of percentage of KG-1 cells and THP-1 cells in media that had survived SMS121 treatment versus DMSO control. Normal distribution was assessed by QQ-plot.

## Results

### Fatty acids are transferred from adipocytes to AML cells

**Figure 1:**
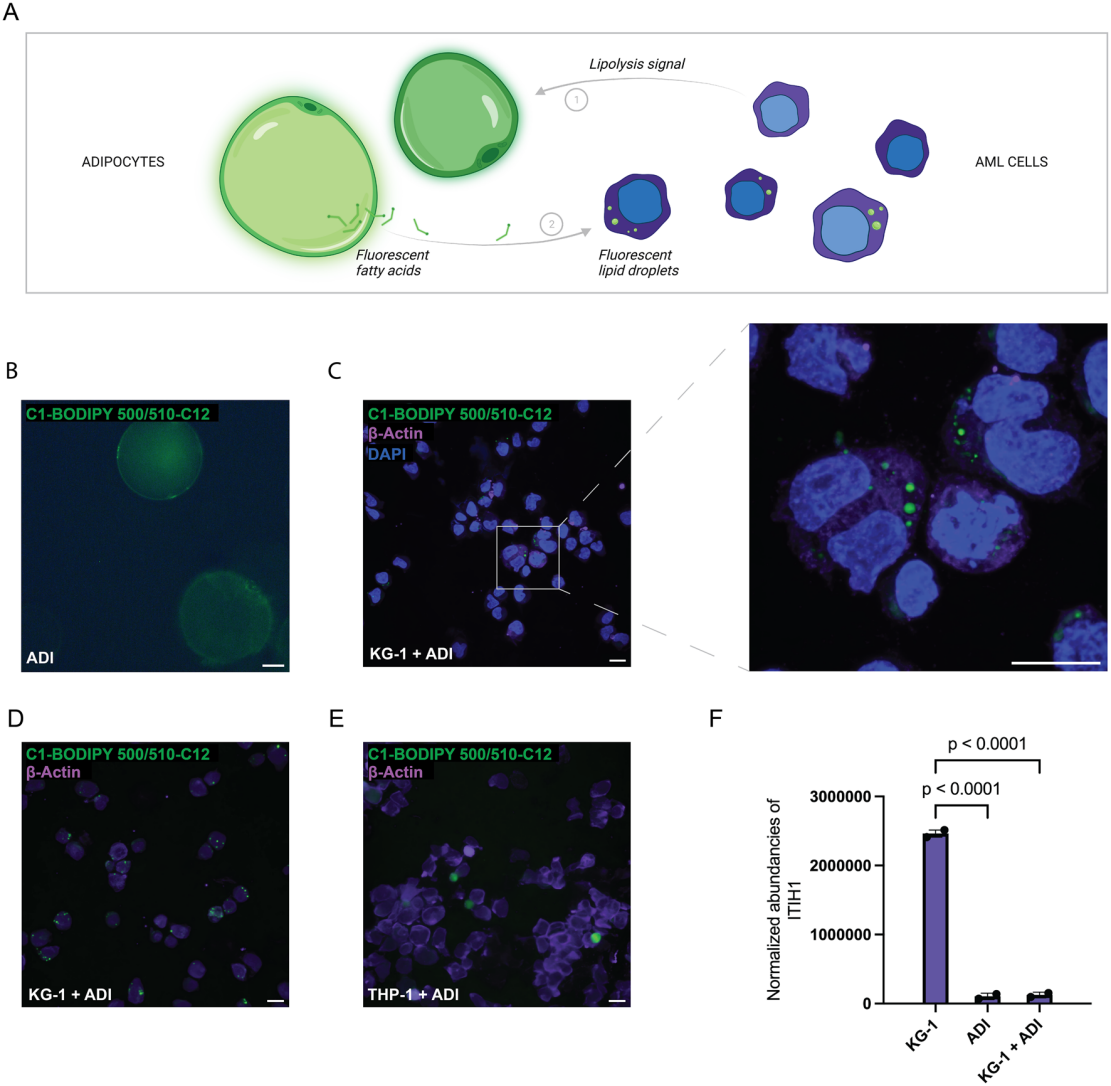
Fatty acids are transferred from human adipocytes to AML cells upon co-culturing. (**A**) Schematic representation of adipocytes loaded with fluorescent fatty acid analogue and subsequently co-cultured with AML cells which leads to transfer of fatty acids to AML cells seen in the form of fluorescent lipid droplets. Created with BioRender.com. (**B**) Adipocytes (ADI) filled with green, fluorescent fatty acid analogue C1-BODIPY 500/510-C12. Image taken with an Axiovert 200 (Zeiss) scale bar is 20 µm. (**C**) Microscopy images of KG-1 cells with uptake of fluorescent lipid after 22 h of co-culture with adipocytes (ADI) pre-loaded with green, fluorescent fatty acid analogue. Nuclei are stained with DAPI (blue) and cell body with actin (violet). Magnification is 40 × without and with (zoomed in) Nyquist sampling. (**D**) KG-1 and (**E**) THP-1 cells after 24-h co-culture with adipocytes (ADI) filled with green, fluorescent fatty acid analogue. Actin staining in violet. Confocal microscopy images (**C**–**E**) are taken with a Nikon A1 plus confocal microscope and are shown as maximum intensity projections with 20 µm scale bars. (**F**) Mass-spectrometry analysis of media from 20-h co-culture of KG-1 cells and adipocytes (KG-1 + ADI) or from media of KG-1 or adipocytes cultured alone. Bars show mean of n = 2, error bars show SD. One-way ANOVA test was performed with the Tukey’s multiple comparisons test. Mean difference between KG-1 and ADI was 2,357,386 95% CI (2,127,726, 2,587,046), p < 0.0001, and between KG-1 and KG-1 + ADI 2,337,043 95% CI (2,107,384, 2,566,703), p < 0.0001. Mean difference between ADI and KG-1 + ADI was not significant with a mean difference of − 20,343 95% CI (− 250,002, 209,317), p = 0.9290.

AML cells in the bone marrow have high energy requirements to satisfy their increased proliferation and it has been shown that malignant cells can induce lipolysis in adipocytes in patients with AML^5^, but the molecular mechanism is unknown (Fig. 1A). Here, we set out to investigate this interplay between primary adipocytes and AML cells. Human primary adipocytes were loaded with C1-BODIPY 500/510-C12 fluorescent fatty acid analogue, which including the fluorophore results in an 18-carbon long-chain fatty acid (Fig. 1B; Supplementary Fig. 1). The adipocytes were co-cultured with AML cells (KG-1 and THP-1) for approximately 24 h. The two cell lines were selected based on that we previously discovered that KG-1 has low expression of the glucose transporter 1 (GLUT1) and likely is less dependent on glycolysis, while THP-1 has high expression of GLUT1 and was subsequently significantly affected by blocking the activity of GLUT1^17^. As can be seen in Fig. 1C, fluorescent lipid droplets were detected in KG-1 cells confirming that fluorescent long-chain fatty acids had been transferred from the adipocytes to the KG-1 cells (Fig. 1C). A significant amount of small fluorescent lipid droplets was detected in most of the KG-1 cells (Fig. 1D). In contrast, only blurry green dots were detected in THP-1 cells, which most likely is a microscopy artefact, as it was also detected in negative samples without pre-stained lipids added, suggesting that less lipids are transferred to the THP-1 cell line (Fig. 1E; Supplementary Fig. 2). To investigate the putative molecular mechanism behind this interplay, we analyzed the media in which the cells had been co-cultured by mass spectrometry to resolve potential proteins that could result in induction of lipolysis in adipocytes. In the media where KG-1 cells had been cultured, there was a significant increase (p < 0.0001) of Inter-alpha-trypsin inhibitor heavy chain 1 (ITIH1) as measured by mass spectrometry (Fig. 1F, Supplementary Tables S1–S3). In contrast, very low amounts of ITIH1 were detected in the co-cultured media, as well as in the media where the adipocytes had been cultured alone (Fig. 1F). ITIH1 is a secretory protein that is known to bind to hyaluronan surrounding adipocytes and induce systemic insulin resistance^18^, which is closely associated to induction of adipocyte lipolysis. Another protein found in KG-1 media that was reduced in adipocyte and co-culture media, however with less variation, was Hornerin (Supplementary Fig. 3). Hornerin has been suggested to contribute to development of malignancy in AML^19^ but no role in adipocytes has been reported, thus we conclude that ITIH1 is more likely to potentially interfere with adipocyte function.

### Expression of CD36 contributes to enhanced fatty acid uptake in AML

**Figure 2:**
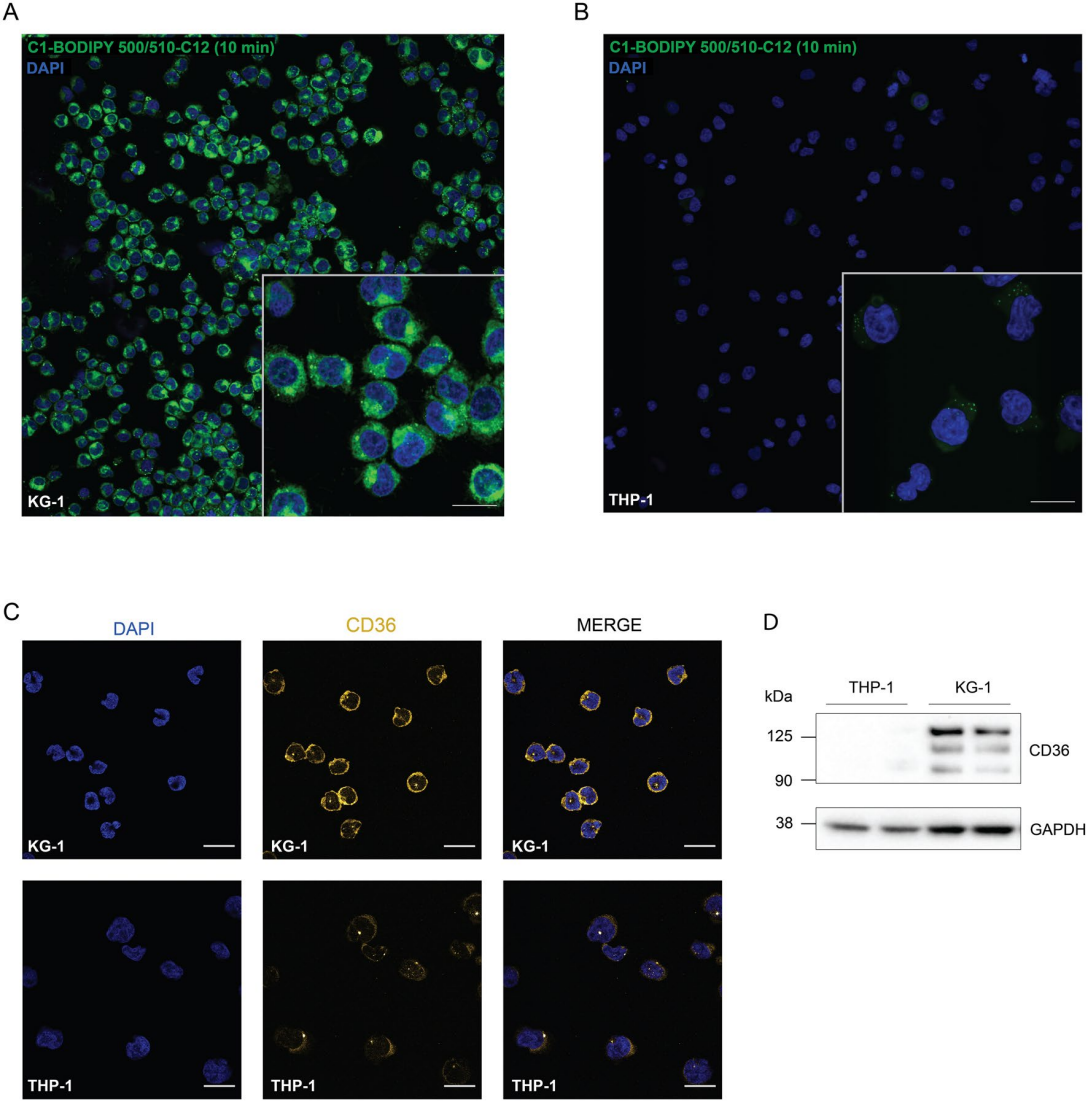
The level of CD36 expression in AML cells affects lipid uptake. Uptake of green, fluorescent fatty acid C1-BODIPY 500/510-C12 after 10 min exposure in (**A**) KG-1 and (**B**) THP-1. Nuclei are shown in blue. Magnification is 20 × without and with (zoomed in) Nyquist sampling. Scale bars show 20 µm. (**C**) Images of CD36 expression in KG-1 and THP-1 cells shown in orange. Nuclei are stained with DAPI (blue). Images were taken with a 60 × objective and scale bars indicate 30 µm. Confocal microscopy images (**A**–**C**) are taken with a Nikon A1 plus confocal microscope. (**D**) Western blot of CD36 expression in THP-1 and KG-1 in duplicate samples. GAPDH was used as loading control. Upper part of the membrane was incubated with anti-CD36, 130 s exposure time. Lower part was incubated with anti-GAPDH with 10 s exposure time.

To clarify the molecular basis behind the differences observed between KG-1 and THP-1 cells related to the transfer of fatty acids from primary adipocytes (Fig. 1D,E), serum starved KG-1 and THP-1 cells were exposed to the BODIPY long chain fatty acid analogue for 10 min and the amount of lipid uptake in the cells were analyzed with fluorescence microscopy. Clearly, the KG-1 cells had higher uptake of the fatty acid analogue compared to THP-1 (Fig. 2A,B, Supplementary Fig. 4). As CD36 is known to be a lipid translocase that facilitates the transfer of long and very long chain fatty acids over the plasma membrane, the expression levels of CD36 were analyzed in the cell lines. Applying confocal microscopy, KG-1 cells showed a high expression of CD36, while the expression was much lower in THP-1 cells (Fig. 2C; Supplementary Fig. 5). To confirm this, the cell lysates were analyzed by western blotting showing clear expression of CD36 in KG-1 cells but only weak staining in the THP-1 cells (Fig. 2D, (Supplementary Fig. 6). This suggests that high expression levels of CD36 in AML cells contribute to an increase in fatty acid uptake.

### Receptor-based virtual screening identified CD36 inhibitors

**Figure 3:**
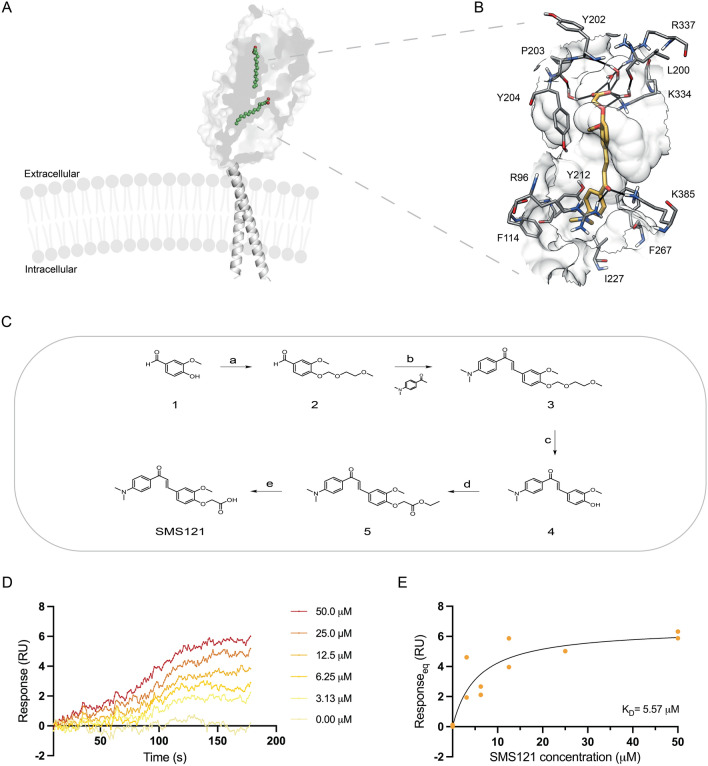
SMS121 binds to CD36 (**A**) Three-dimensional structure of CD36. The figure was made in Pymol by aligning CD36 model (PDB ID: 5LGD) and AlphaFold generated CD36 model. The extracellular domain with two fatty acids colored in green in the cavity is shown as surface while the transmembrane domain from AlphaFold CD36 model is present as cartoon. Cell membrane was created with BioRender.com. (**B**) Minimized average structure of CD36 in complex with the inhibitor (SMS121) shown in orange identified through virtual screening. The ligand–protein and ligand–water–protein H-bonds are highlighted as black lines. (**C**). Steps for synthesis of SMS121. Reagents and conditions: (a) NaH, 2-Methoxyethoxymethyl chloride, THF, RT, 2 h; (b) NaOH (s), MeOH, RT, 16 h; (c) HCl 1 N, MeOH, 75 °C, 1.5 h; (d) anhydrous K_2_CO_3_, dry DMF, ethyl bromoacetate, RT, 16 h; (e) aq. LiOH 2 N, MeOH/THF (1:1 v/v), RT, 2 h. (**D**) The responses for SMS121 samples injected over immobilized CD36. SMS121 concentrations shown are 0–50 μM. (**E**) The response values for SMS121 plotted against concentration using the average response in the last 10 s of association.

With the aim of identifying novel CD36 inhibitors, we developed a receptor-based virtual screening (VS) study focused on the X-ray structure of CD36 in complex with two palmitic acid molecules (PDB code: 5LGD)^14^. which was used as a reference (Fig. 3A). By inspecting the X-ray structure, as reported by Higgins and co-workers, it is possible to see that the CD36 protein is characterized by a central cavity mainly delimited by hydrophobic side chains that has a key function in the fatty acid uptake performed by CD36. Inside this cavity, two fatty acids are present and lie along the length of the cavity (Fig. 3A). Based on these observations, we applied a consensus docking strategy^15^ for the identification of new ligands able to interact into the CD36 cavity, as this computational approach was demonstrated to represent a profitable strategy for identifying new hit compounds through VS studies^20,21^. By using this method, each ligand is docked into the target protein by means of different docking procedures. Then, among the different top-scored poses (originated by the different docking procedures), the pose predicted by the largest number of docking procedures is considered as the best docking pose. Starting from a pre-filtered database of about 32,000 molecules, we performed a thorough docking-based strategy in which 12 different docking procedures were employed. The results obtained from the consensus docking analysis, reported in Table 1, showed that no ligand achieved full consensus among all the docking procedures, since a maximum consensus level of 9 was obtained for the compounds. These results agree with our previous VS studies based on consensus docking in which we observed that, statistically, only a very small percentage of the analyzed compounds is able to reach a high consensus among many different docking procedures and often no compound obtains the maximum consensus level achievable^22–24^. Based on the results produced by the consensus docking protocol herein reported, six molecules showing at least a consensus level of 8 were selected for the next evaluation step.

**Table 1:** Consensus docking results.

Consensus level	No. of compounds
12	0
11	0
10	0
9	1
8	5
7	64
6	249
5	934
4	1720
3	6018
2	15,830
1	7049

### Molecular dynamics simulations predict a stable binding to the central cavity of CD36

The six CD36-ligand complexes generated by the docking experiments were subjected to a 100 ns molecular dynamics (MD) simulation protocol (see Methods section for details) aimed at evaluating the strength of the ligand–protein interactions. The results were analyzed in terms of percentage of H-bond stability during the simulation. All analyzed compounds showed strong H-bond interactions with residues K334 and R337 and were thus visually checked and analyzed based on their similarity. Four different clusters were identified (Supplementary Fig. 7) and among those clusters, three corresponded to molecules that are able to only interact at the entrance of the cavity; whereas compound four (SMS121) was the only one that was deeply inserted inside the cavity and was therefore selected for further analyses (Supplementary Fig. 7D). In particular, the carboxylic group of the ligand shows a direct ionic interaction with K334, three water-mediated interactions with the backbone oxygens of L200, Y202 and P203, and a fourth water-mediated interaction with the sidechain of R337, whereas the sidechain of Y204 shows lipophilic interactions with the 2-methoxyphenoxy fragment of the ligand (Fig. 3B). The carbonyl oxygen of the ligand shows H-bond interactions with R96 and K385, whereas the *N*,*N*-dimethyl aniline fragment shows lipophilic interactions with F114, Y212, I227 and F267.

### The SMS121 compound binds to CD36 with low micromolar affinity

To experimentally confirm the results from the virtual screening study, compound SMS121 was synthesized (Fig. 3C) and binding studies were conducted by surface plasmon resonance using a BIAcore® 3000 instrument. CD36 was immobilized on the chip and the SMS121 compound was run as analyte at different concentrations (0–50 μM). As can be seen in Fig. 3D, binding curves were recorded although at low response unit (RU) levels. The low RU levels is likely due to that SMS121 is rather small compound (MW = 355 Da), which is at the verge of what a Biacore® 3000 instrument can detect (Special Features of Biacore® Instrumentation—Biosensor Core). Still, the K_D_ could be calculated based on plotting the concentration of SMS121 against the RU value to be approximately 5 µM (Fig. 3E).

### SMS121 impairs lipid uptake and reduce the viability in AML cells

**Figure 4:**
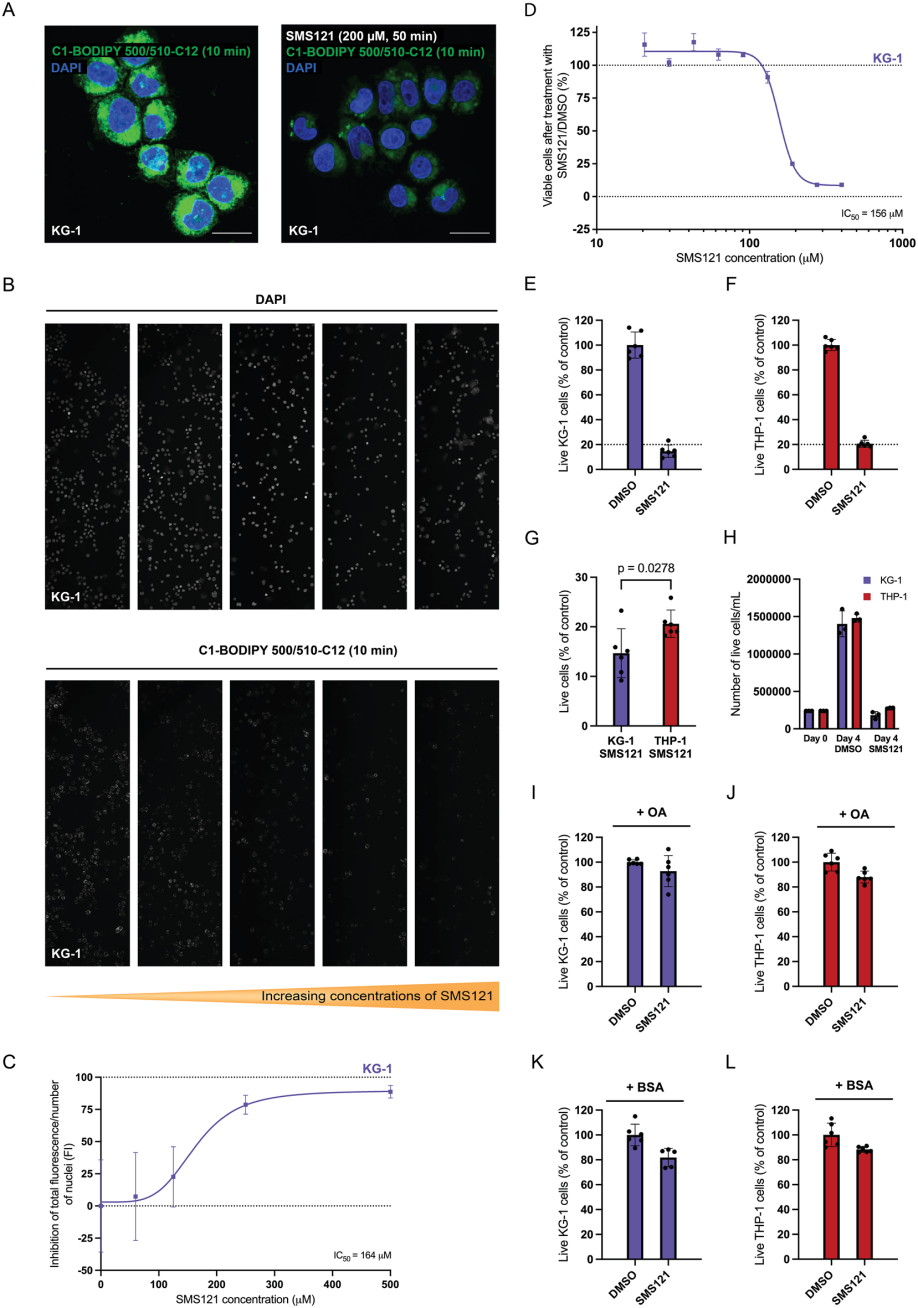
SMS121 inhibits lipid uptake and affects viability of AML cells. (A,left) KG-1 cells exposed to green fluorescent fatty acid analogue C1-BODIPY 500/510-C12, (**A**, right) in the presence of inhibitor SMS121. Inhibition with 200 µM SMS121 was performed for 50 min followed by additional 10 min of incubation together with C1-BODIPY 500/510-C12. Images are taken with a Nikon A1 plus confocal microscope and are shown as maximum intensity projections. Magnification is 40 × with Nyquist sampling. Scale bars are 20 µm. (**B**) Concentration dependent inhibition of green fluorescent lipid by SMS121. Representative figures from three samples per condition of KG-1 cells showing nuclei (DAPI) and lipid uptake of C1-BODIPY 500/510-C12 for 10 min in KG-1 cells that had been pre-incubated for 50 min with increasing concentrations of SMS121 from left to right (concentration of SMS121 = 0, 62.5, 125, 250, 500 µM). Magnification is 10×. (**C**) The inhibition of fluorescent lipid uptake in figure C per number of nuclei with an increasing concentration of SMS121. Dots are mean of n = 3 samples with error bars as SD. Curve shows a non-linear regression with IC_50_ calculated to 164 µM SMS121. (**D**) ATP-based cell viability assay in KG-1 cells showing viability of cells after 72 h of SMS121 addition in concentrations from 0 to 400 µM in comparison to DMSO control. The IC_50_ value is 156 µM. Error bars show SD, n = 3. (**E**, **F**) Live cell count by trypan blue exclusion after 96 h of treatment with 150 µM SMS121 in comparison to DMSO controls in KG-1 (14.7% mean survival, violet) and THP-1 (20.6% mean survival, red). Bars show mean of n = 6, error bars show SD. (**G**) Two-tailed unpaired t-test of the survival in E and F resulted in mean difference of -5.93 95% CI (− 11.06, − 0.79), p = 0.0278. (**H**) Number of live cells/mL seeded out on day 0 and after 4 days of 150 µM SMS121 or DMSO treatment in KG-1 and THP-1 cells. Bars show mean of n = 3, error bars show SD. (**I**–**L**) The live cell count trypan blue test was also performed on cells grown in (**I**, **J**) media supplemented with 150 µM oleic acid (+ OA) or (**K**, **L**) media supplemented with 850 µg BSA (+ BSA).

To analyze if the CD36 inhibitor blocks the uptake of lipids into AML cells, serum starved KG-1 cells (selected as they have high expression of CD36) were exposed to the BODIPY fluorescent fatty acid analogue in the presence or in the absence of SMS121. In cells that had not been exposed to the inhibitor prior to the addition of pre-stained lipid, clear fluorescent staining from the BODIPY fluorescent fatty acid analogue could be detected (Fig. 4A, left), while in cells that were exposed to SMS121 in addition to the BODIPY fluorescent fatty acid analogue, very low amounts of staining could be detected by microscopy (Fig. 4A, right, Supplementary Fig. 8), showing that the inhibitor blocks the uptake of fatty acids into the AML cells. To quantify the effect SMS121 compound has on lipid uptake, the IC_50_ value was calculated. Thus, the change in fluorescence in KG-1 cells exposed to pre-stained lipid and increasing concentrations of SMS121 (0–500 µM) was followed by microscopy (Fig. 4B). The concentrations were plotted against the fluorescence and the IC_50_ was calculated to be 164 µM (Fig. 4C). To determine the potency of the compound the KG-1 cells were exposed to different concentrations of SMS121 (0–400 µM), and cytotoxicity was measured with a cell viability assay. As can be seen in Fig. 4D, SMS121 affects the viability of KG-1 cells and the IC_50_ was calculated to be 156 µM (Fig. 4D). Interestingly, the potency of the SMS121 compound was similar in THP-1 cells (161 µM, Supplementary Fig. 9), suggesting that the effect is not strictly dependent on the amount of CD36 expression. Based on these IC_50_-values, both KG-1 and THP-1 cells were treated with 150 µM SMS121 and the number of live cells after 4 days were calculated. An evident reduction (14.7% viable cells) in the amount of viable KG-1 cells was detected after addition of SMS121 (Fig. 4E). Likewise, the viability of THP-1 cells was also affected by SMS121 (20.6% viable cells), but to a significantly lower extent (p = 0.0278) compared to KG-1 (Fig. 4F,G). The number of live cells is constant upon SMS121 treatment for 4 days, while control cells (DMSO treated) continued to proliferate and reached a six times higher cell density (Fig. 4H). The significant difference detected between the cell lines could be explained by THP-1 having a more glycolytic growth dependence and would therefore be more affected by blocking uptake of glucose than fatty acids, which has also previously been observed^17^. However, the difference between KG-1 and THP-1 is not as prominent as in the acute lipid uptake experiments, suggesting that long-term effects are not as dependent on the number of CD36 receptors expressed on the cell surface. To verify that it is targeting of CD36 that is affecting the viability of cells, rescue experiments were executed by applying either an 18-carbon fatty acid and/or BSA (BSA has previously been shown to bind to the same pocket on CD36 as long chain fatty acids^25^). Clearly, AML cells co-treated with SMS121 and fatty acid and/or BSA are rescued, as the viability is at similar levels as for untreated cells (Fig. 4I–L).

## Discussion

Fatty acid oxidation is a crucial catabolic pathway allowing for abnormal cell proliferation in AML^26^. Chemotherapy-resistant AML cells exhibit increased oxidative phosphorylation dependency accompanied by increased fatty acid oxidation and upregulated expression of the fatty acid transporter CD36^27^. Consequently, given the biological role of lipid metabolism in AML development and in chemotherapy resistant leukemic relapse, there is an urgent need to better understand underlying molecular mechanisms and explore lipid uptake as a therapeutic target. Here we report the development of a small molecule compound targeting CD36 which affects both the uptake of lipids as well as the viability of AML cells.

CD36 is an integral membrane protein (MW ~ 88 kDa) with a large extracellular domain (MW ~ 47 kDa) and two transmembrane spanning helices^14^. CD36 is a fatty acid translocase that facilitates the transport of long and very long chain fatty acids over the membrane, accounting for 50% of the FA uptake^8,28^, while short- and medium-chain fatty acids (under C_12_) can passively diffuse across the cell membrane. CD36 has been identified as a new promising therapeutic target for cancer, since it strengthens metastasis initiation potential of cancer cells and upon blocking its lipid uptake activity, with a CD36 specific antibody, the size of lymph node metastases is reduced by 80–90%^29^. Moreover, reports have shown that high expression of CD36 contributes to chemotherapy resistance in leukemia^7,27^ and correlates with poor survival of patients with AML^11^ as well as in lung, bladder and breast cancer^29^. In addition, cancer cells can undergo metabolic adaptation, such as increased expression of CD36 to leverage on the adipose microenvironments, leading to high levels of fatty acid uptake and oxidation, besides resulting in chemotherapy resistance^7^.

A recent report highlighted CD36 as a driver of leukemic blast metastasis in AML, showing CD36 expressing blasts being largely enriched after chemotherapy and maintaining their migratory ability^10^. The authors conclude that CD36 is dispensable for lipid uptake, however the study was conducted in normal media (not starved cells), which agrees well with our results showing that there is no difference in acute lipid uptake in high and low CD36 expressing cells in normal media (Supplementary Fig. 10). Moreover, inhibition of CD36 in a xenograft mouse model reduced metastasis of blasts and prolonged survival of chemotherapy treated mice^10^. The bone marrow niche is critical for AML development^3^, and AML blasts have been shown to directly induce lipolysis in adipocytes resulting in the transfer of fatty acids from adipocytes to AML blasts^5^. A similar observation has been done in chronic lymphocytic leukemia (CLL) patients, where high amounts of the lipid oleoylethanolamide (OEA) was detected with mass spectrometry in plasma and OEA was suggested to be a lipolytic factor produced by CLL cells to fuel their growth^30^. Here we detected high amounts of ITIH1 in the AML cell media. ITIH1 is a secretory protein that upon overproduction cause systemic insulin resistance^18^, which is closely associated with adipocyte lipolysis. However, more research is needed to confirm ITIH1’s involvement in fatty acid release. Still, as the presence of adipocytes in the co-cultures with AML cells resulted in significant decreased levels of ITIH1 in the media, and since ITIH1 was previously shown to interact with adipocytes^18^, we speculate that ITIH1 could contribute to AML induced lipolysis in adipocytes.

As myeloid blasts develop in the bone morrow, and the volume of the bone marrow consists of up to 70% adipocytes, metabolic adaptation towards increased fatty acid oxidation in AML has been suggested to be a vulnerability that could be targeted. This was applied by Tcheng and co-workers who discovered that very long chain fatty acid metabolism is critical to AML cell survival and that targeting the enzyme that metabolizes fatty acids results in AML cell death^9^. Furthermore, knock-down of the very long chain acyl-CoA dehydrogenase (ACADVL) or small molecule inhibition of MCL-1, a protein that interacts with ACADVL, restored sensitivity to Venetoclax/Azacitidine in resistant cells^31^. In addition, targeting another enzyme involved in the entry of fatty acids into the mitochondria (CPT-1), resulted in inhibition of fatty acid oxidation and induction of cytotoxicity in AML^26^. Here we present an alternative approach to block fatty acid oxidation in AML, namely by targeting the first step in the fatty acid oxidation—the fatty acid transporter CD36, with a drug-like small-molecule compound. CD36 specific antibodies have previously successfully been used as therapeutics in animal models^29^, and sulfo-N-succinimidyl derivatives of long-chain fatty acids (e.g. SSO, a derivative of oleate) has been shown to block the lipid uptake activity at micro molar concentrations^32^, but as small molecule compounds are more economically sustainable than biologics and easier to access for patients, there is a clear need to develop drug-like small molecules that can be used in therapy. As the three-dimensional structure of the CD36 ectodomain has been determined in complex with a long chain fatty acid^14^, we utilized this information to perform a virtual screening study aiming at identifying a novel CD36 inhibitor. The most promising compound suggested by the modeling studies was experimentally validated and shown to block the activity of CD36 by decreasing the amounts of long chain fatty acids that could be taken up by AML cells, and additionally generating reduced AML cell viability. It should be noted that fatty acid uptake was analyzed with acute experiments (minutes) in AML cells starved from FBS, while the viability experiments were long term experiments (days) in the presence of 2–10% FBS. The increased amount of CD36 in KG-1 cells compared to THP-1 impacted acute lipid uptake experiments massively, while the difference in effect on the long-term experiments on cell viability was more moderate, suggesting that the amount of CD36 is not directly proportional to the viability. However, the viability for KG-1 cells was still significantly more affected than for THP-1 cells. Together, this indicates that blocking CD36 during long-term is dependent on CD36 expression per se, not the amount of expression which seem to mainly affect the acute experiments. Moreover, as albumin can bind to the CD36 receptor^25^, the addition of BSA (or FBS including BSA) rescued AML cells from the SMS121 effect, and largely reduced the amount of fluorescent fatty acids taken up by KG-1 cells (Supplementary Fig. 10). Long-term effects of SMS121 on the viability could also be reversed by addition of oleic acid. Hence, potential protecting effect from natural levels of albumin surrounding AML cells in blood and bone marrow must be taken into consideration for targeting CD36 in patients, and therefore there is a need to improve the affinity of SMS121 for CD36 before it can be judged as a lead compound and validated for AML treatment. SMS121 most likely works in an antagonistic manner by occupying the cavity where the lipid is binding, resulting in decreased lipid uptake. However, as CD36 is a scavenger receptor and has been reported to be for instance of importance of monocyte differentiation^33^, other molecular mechanisms than lipid uptake may also be affected upon treatment with CD36 inhibitors. Thus, at this stage we cannot exclude potential off-targets effects by SMS121, but we can conclude that it targets CD36, inhibits CD36 activity (inhibit lipid uptake) and decreases the viability of AML cells. Moreover, to elucidate the exact processes mediating the reduction in the number of AML cells, e.g. if the cells undergo apoptosis upon SMS121 treatment or if the cells simply are metabolically affected and therefore reduce their proliferation rate, more studies are needed.

## Conclusions

Fatty acid oxidation is a central catabolic pathway allowing for abnormal cell proliferation in AML. In addition, chemotherapy-resistant AML cells exhibit increased oxidative phosphorylation accompanied by upregulation of the fatty acid transporter CD36. Thus, the metabolic adaptation towards increased fatty acid oxidation in AML is a vulnerability that could be targeted. Here we present a drug-like inhibitor providing a blueprint for further compound development and drug discovery targeting CD36, a central molecule in lipid metabolic reprogramming of cancer cells.

### Supplementary Information

Supplementary Figures.

Supplementary Tables.

## Data Availability

For original data, please contact: karin.lindkvist@med.lu.se or giulio.poli@unipi.it.
