# Supplementary material for: SMS121, a new inhibitor of CD36, impairs fatty acid uptake and viability of acute myeloid leukemia
Source: Sci Rep. 2024 Apr 20;14:9104. doi: 10.1038/s41598-024-58689-1 (PMC11032350; doi:10.1038/s41598-024-58689-1)
Supplement: Supplementary file 1 — Supplementary Figures. [file 41598_2024_58689_MOESM1_ESM.docx]

**Supplemental information for**

**SMS121, a new inhibitor of CD36, impairs fatty acid uptake and viability of acute myeloid leukemia**

Hannah Åbacka^1^, Samuele Masoni^2, 4^, Giulio Poli^2*^, Peng Huang^1^, Francesco Gusso^2^, Carlotta Granchi^2^, Filippo Minutolo^2^, Tiziano Tuccinardi^2^, Anna K Hagström-Andersson^3^, Karin Lindkvist-Petersson^1, 4*^.

^1^ Department of Experimental Medical Science, Lund University, Lund, Sweden.

^2^ Department of Pharmacy, University of Pisa, Pisa, Italy.

^3^ Department of Laboratory Medicine, Division of Clinical Genetics, Lund University, Lund, Sweden.

^4^ LINXS - Institute of Advanced Neutron and X-ray Science, Lund, Sweden

^*^To whom correspondence may be addressed: Prof. Karin Lindkvist-Petersson, Department of Experimental Medical Science, Lund University, BMC C13, 221 84 Lund, Sweden, +46 46 2228041, E-mail: karin.lindkvist@med.lu.se or E-mail: giulio.poli@unipi.it


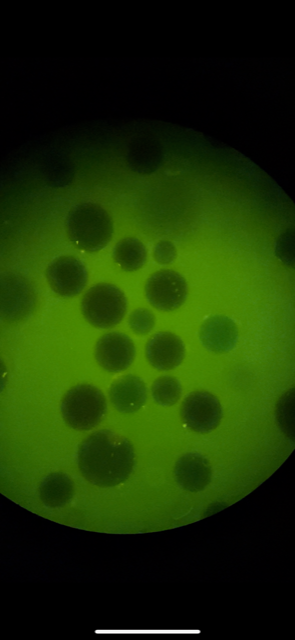

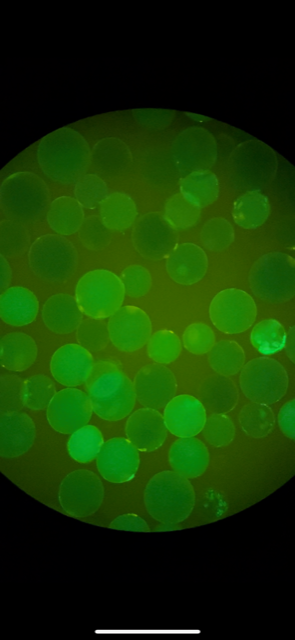


**Supplementary Figure 1.** Human adipocytes after (left) loading with C1-BODIPY 500/510-C12 fluorescent fatty acid analogue and (right) without fatty acid analogue. An Olympus CKX41 microscope with 10x magnification was used.


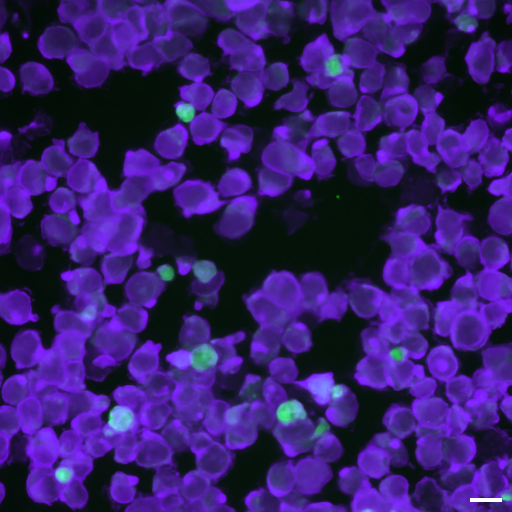


**Supplementary Figure 2.** Negative control of lipid uptake from human adipocytes in THP-1 cells. Magnification is 40x from a Nikon A1 plus confocal microscope. Cells are shown as maximum intensity projections and scalebar is 20 µm.


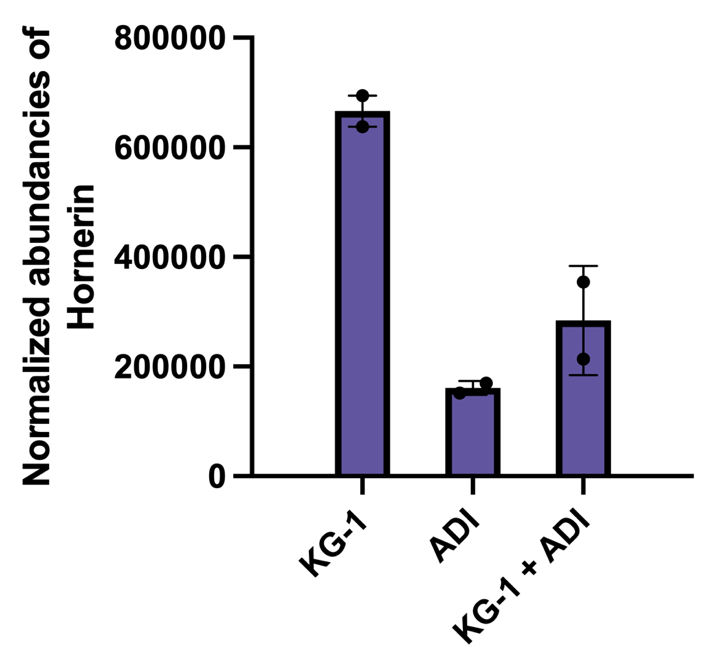


**Supplementary Figure 3.** Amount of Hornerin by mass-spectrometry analysis in media from KG-1 cells, adipocytes (ADI) or KG-1 in co-culture with adipocytes (KG-1 + ADI) for 20 hours. Bars show mean of n = 2 and error bars show SD.


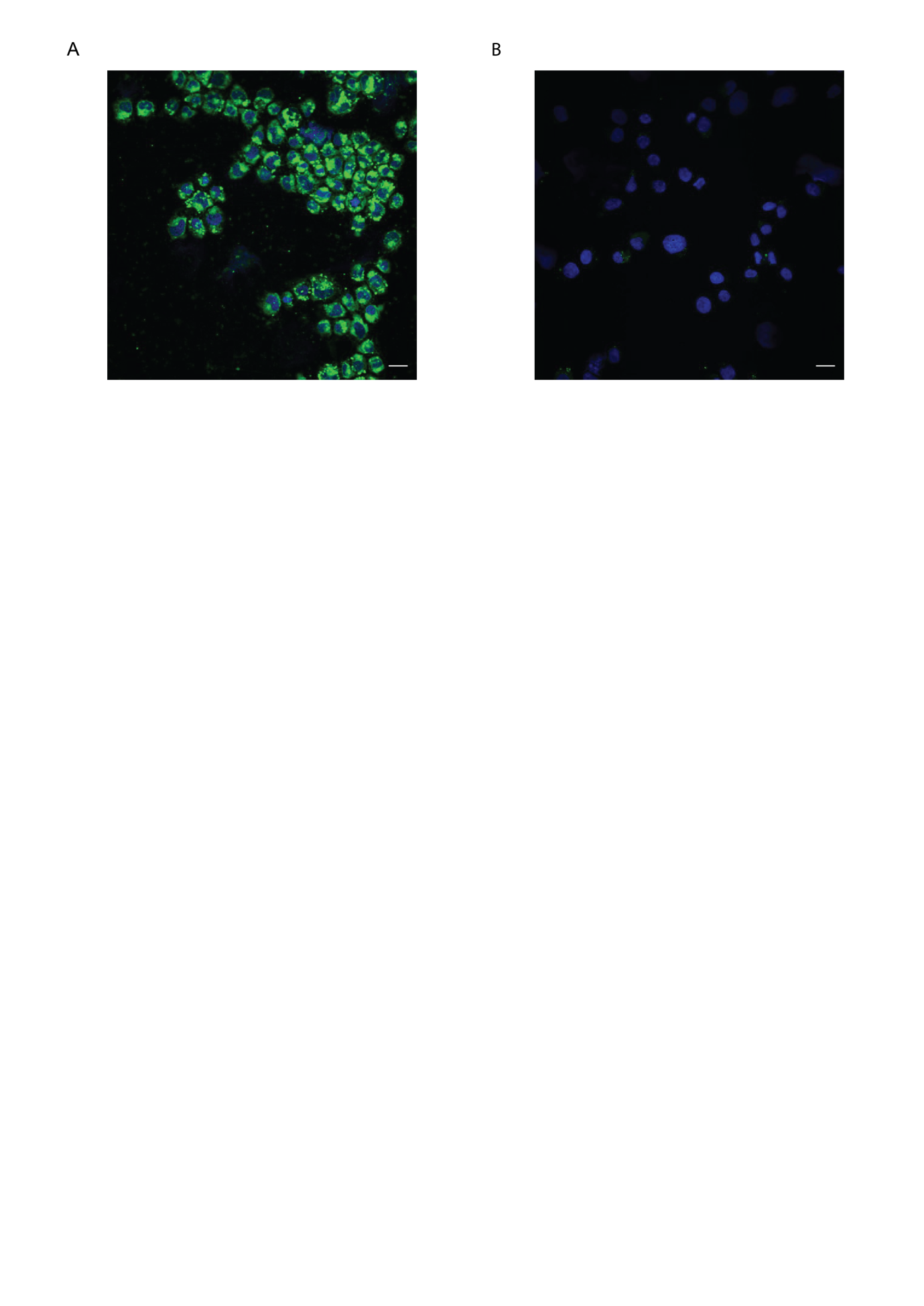


**Supplementary Figure 4.** Repeated analysis of uptake of C1-BODIPY 500/510-C12 fluorescent fatty acid analogue after 10 minutes in (A) KG-1 and (B) THP-1 taken with a Nikon A1 plus confocal microscope. The nuclei are stained with DAPI (blue). Magnification is 40x and the scale bars are 20 μm.


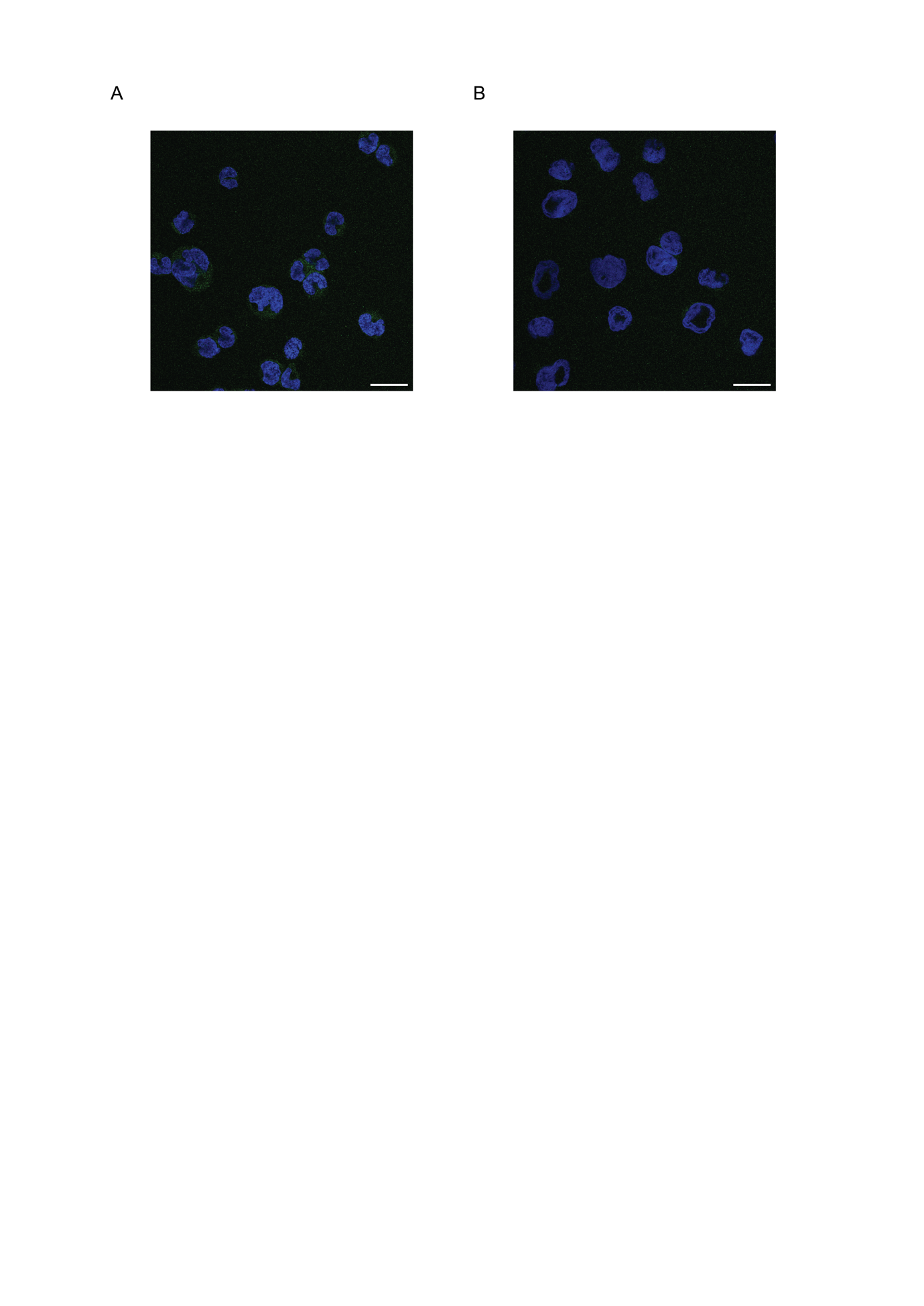


**Supplementary Figure 5.** Negative controls of CD36 (green) expression in AML cells using only secondary antibodies and DAPI (blue). (A) KG-1, (B) THP-1. Magnification is 60x and scale bar represents 30 μm. A Nikon A1 plus confocal microscope was used.

**
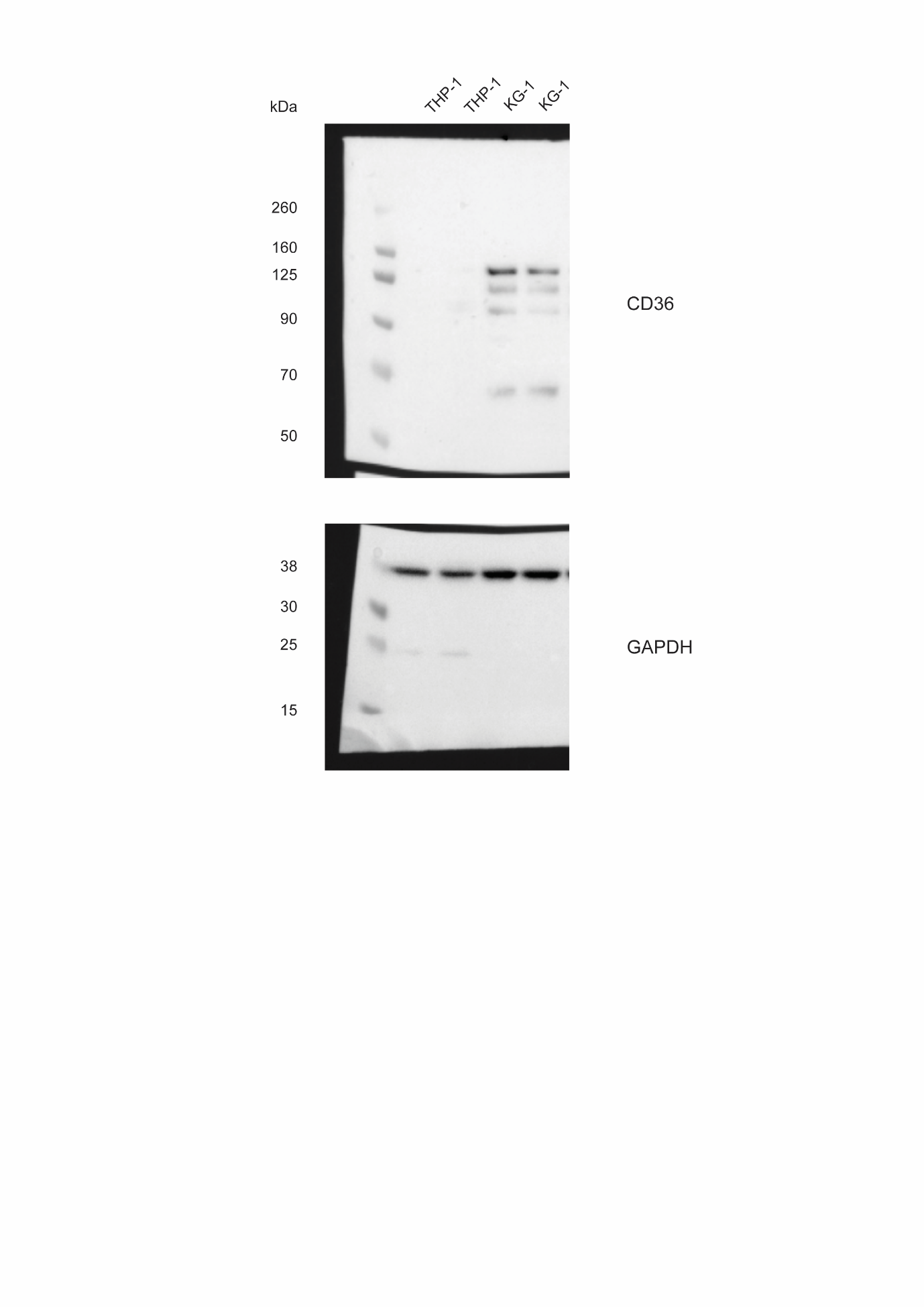
**

**Supplementary Figure 6.** Whole western blot membrane including protein ladder of CD36 expression in THP-1 and KG-1. Upper part of the membrane was incubated with anti-CD36, 130 s exposure time. Lower part was incubated with anti-GAPDH with 10 s exposure time.


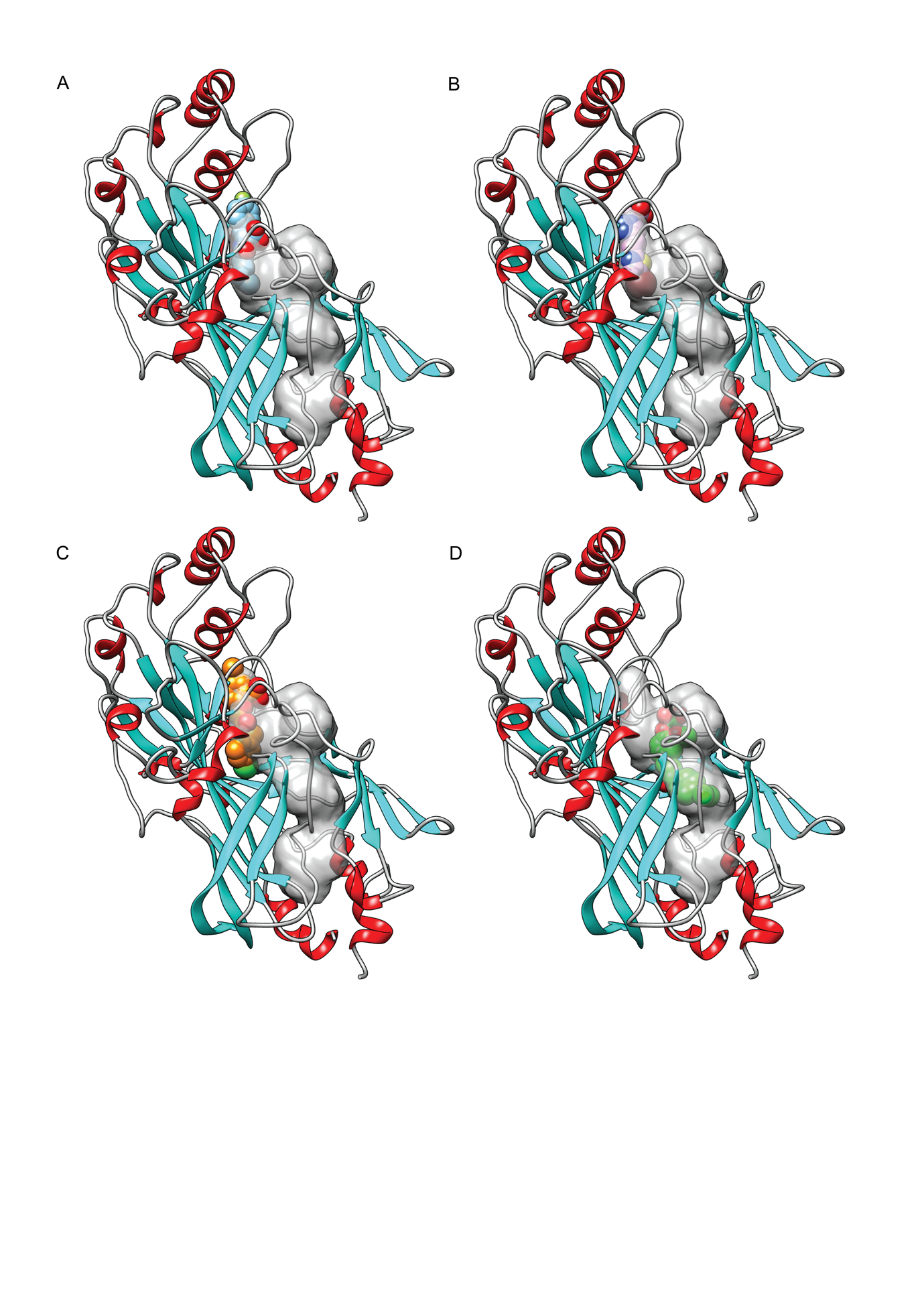


**Supplementary Figure 7.** The four clusters identified by molecular dynamic simulations. (D) Shows SMS121.


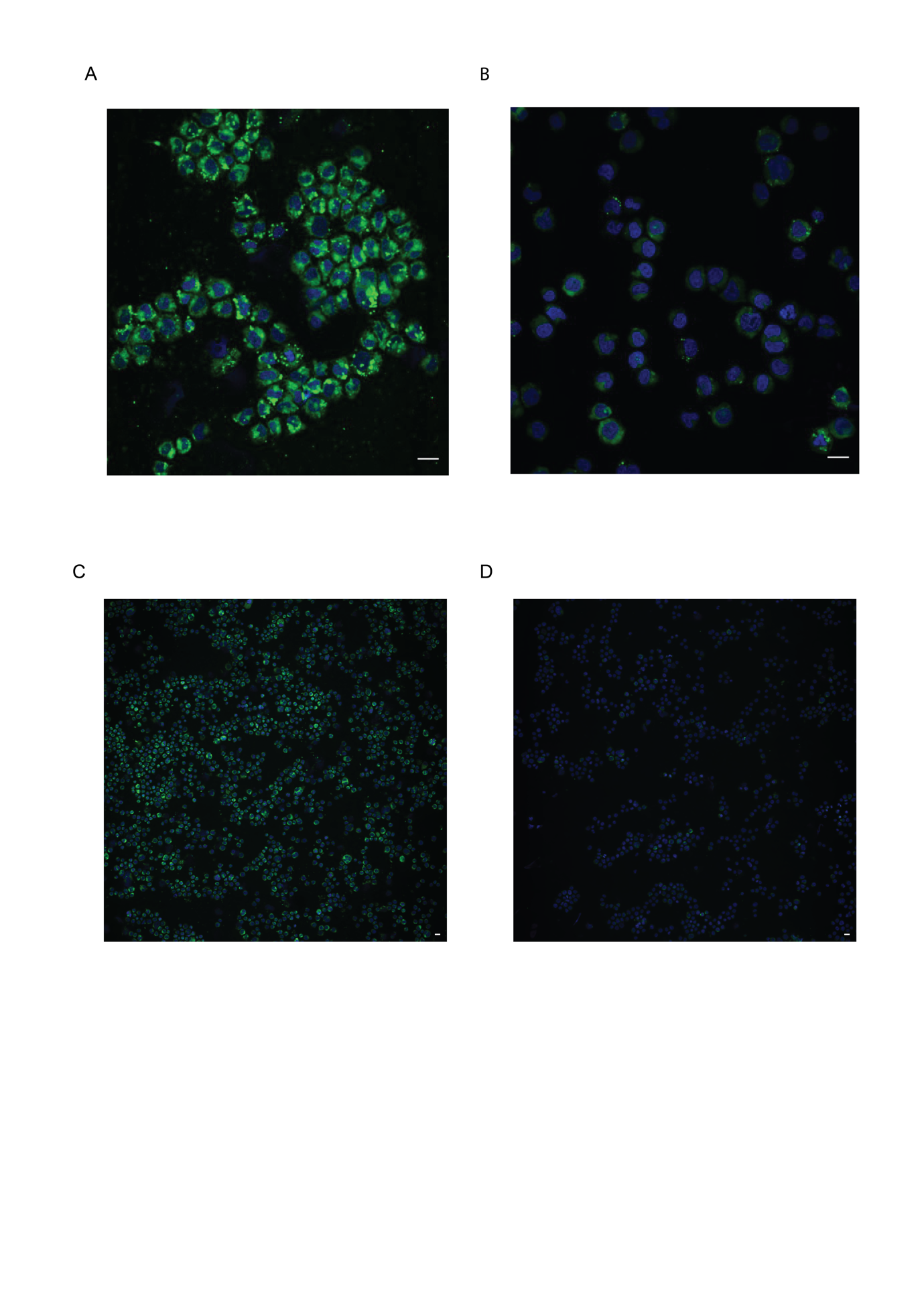


**Supplementary Figure 8.** Two additional analyses of green C1-BODIPY 500/510-C12 uptake in KG-1 cells during 10 minutes (A,C) without and (B,D) with 50 min pre-incubation with 200 μM SMS121. Images are taken with a Nikon A1 plus confocal microscope. Nuclei are stained with DAPI (blue). Magnifications are (A, B) 40x and (C, D) 10x. Scale bars are 20 μm.

**
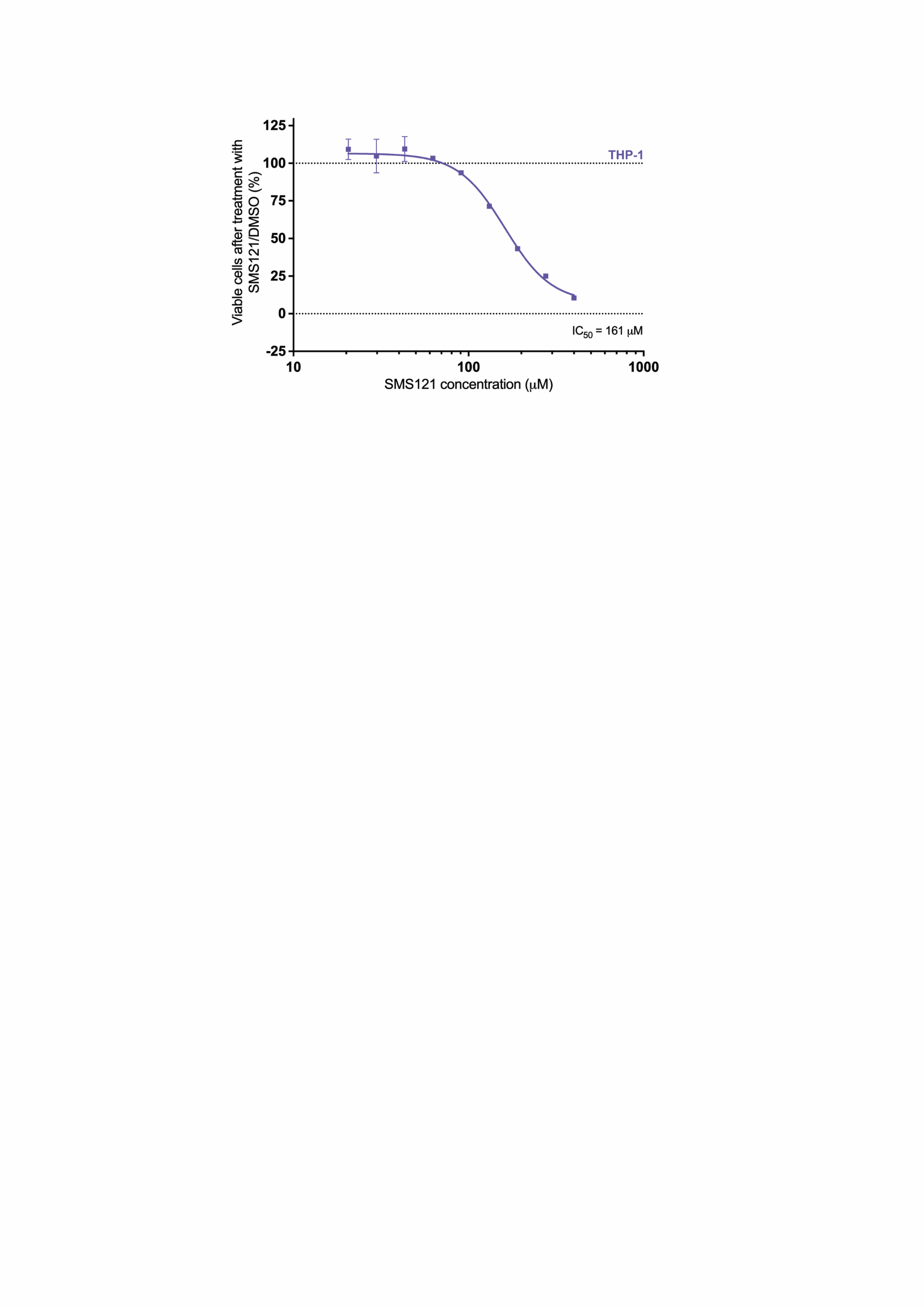
**

**Supplementary Figure 9.** ATP-based cell viability assay in THP-1 cells. Viability of cells is shown after 72 hours of incubation with SMS121 in concentrations from 0 to 400 µM compared to DMSO control. The IC_50_ value is 161 µM. Error bars show SD, n = 3.

**
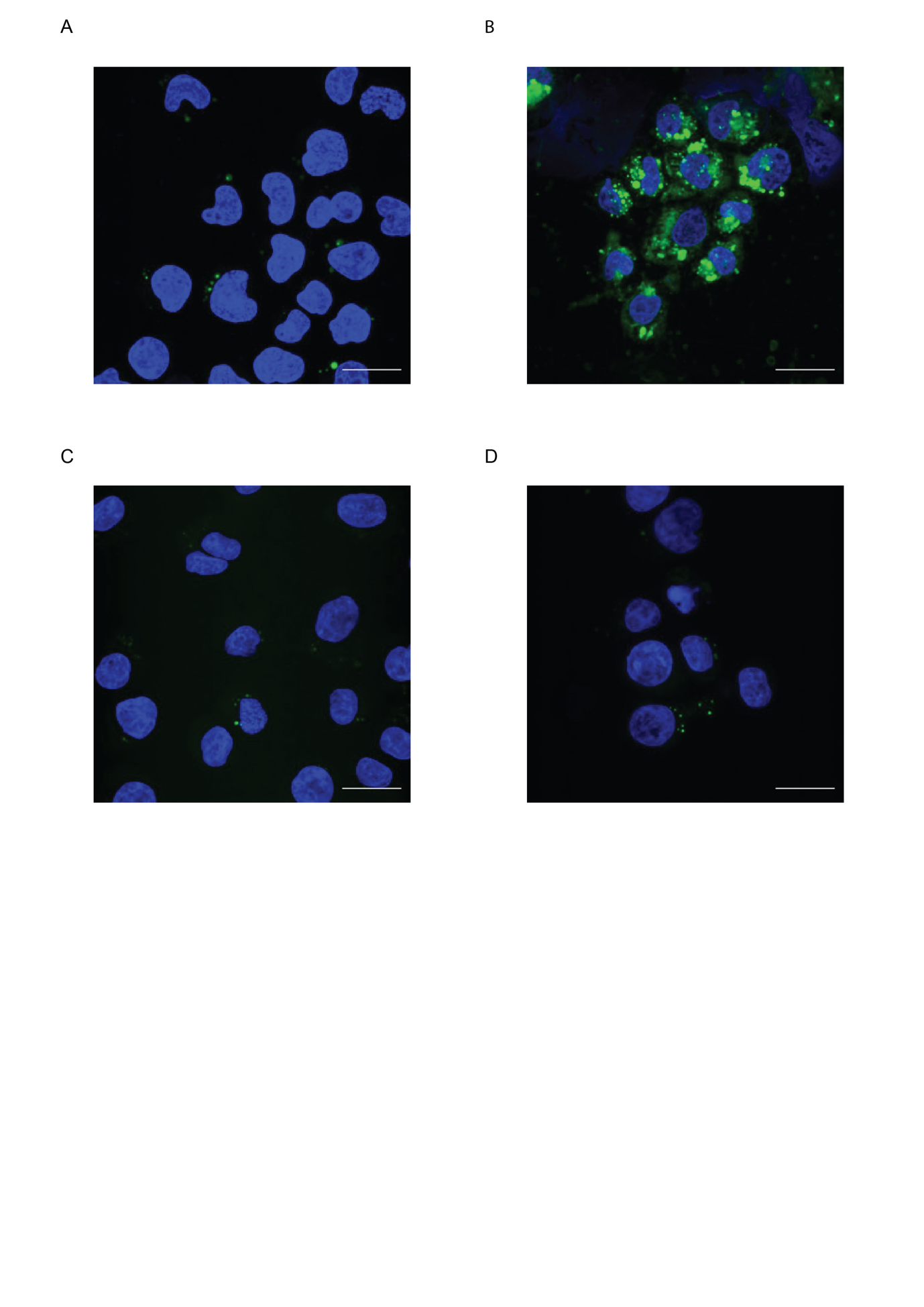
**

**Supplementary Figure 10.** Uptake of green C1-BODIPY 500/510-C12 for 10 minutes. Figures represent (A, B) KG-1 cells and (C, D) THP-1 cells in (A,C) 10 % FBS or (B,D) in 0 % FBS. Images are taken with a Nikon A1 plus confocal microscope. Nuclei are stained with DAPI (blue). Magnification is 40x with Nyquist sampling. Scale bars are 20 μm.
